# Targeting neurobehavioral substrates of effortful control to reduce childhood anxiety: The Camp Kidpower study protocol

**DOI:** 10.21203/rs.3.rs-6342546/v1

**Published:** 2025-10-15

**Authors:** Ka I Ip, Meryl Rueppel, Katherine Raguckas, Jamie Lawler, Kate Rosenblum, Jason Moser, Kate Fitzgerald

**Affiliations:** 1 Institute of Child Development, University of Minnesota; 2 Columbia University, Department of Psychiatry; 3 Eastern Michigan University, Department of Psychology; 4 University of Michigan, Department of Psychiatry; 5 Michigan State University, Department of Psychology

**Keywords:** childhood anxiety, effortful control, ERN, interchannel phase synchrony, RCT

## Abstract

**Background:**

Early childhood anxiety affects up to 20% of preschoolers, with long-term implications including depression, substance abuse, and suicide. Despite some success with exposure-based cognitive behavioral therapy (CBT), many children retain clinically significant symptoms, highlighting the need for innovative interventions. This study integrates the Research Domain Criteria (RDoC) and experimental medicine frameworks to target effortful control (EC)—the capacity to regulate attention and impulses—to reduce anxiety in young children through a novel intervention, *Camp Kidpower*.

**Methods:**

This randomized clinical trial involves 90 preschoolers diagnosed with anxiety disorders, assigned to either an EC training camp or an active comparator condition (child-led play; CLP). The EC intervention incorporates play-based activities targeting selective attention, inhibitory control, and working memory, delivered over five weekly sessions with at-home practice. Anxiety and EC were assessed pre- and post-intervention using clinician ratings, parent/teacher reports, lab-based tasks, and neurophysiological measures (e.g., error-related negativity [ERN] and interchannel phase synchrony [ICPS]). Exploratory analyses include dose-response effects and moderators such as baseline EC, parental psychopathology, and child temperament.

**Discussion:**

*Camp Kidpower* aims to enhance EC to mitigate anxiety in preschoolers, addressing the limitations of traditional CBT by offering an accessible, community-based intervention. This study will provide insight into the neurobehavioral mechanisms linking EC and anxiety, inform the design of targeted treatments, and evaluate potential moderators of intervention efficacy. If successful, the intervention could be disseminated broadly to clinics, schools, and community settings.

Early childhood anxiety is a public health concern that affects up to 20% of preschoolers (Bufferd et al., 2011; Whalen et al., 2017), often persists into adulthood (Keller et al., 1992), and predicts a wide range of negative long-term outcomes, including major depression, school drop-out, substance abuse, and suicide (Beesdo et al., 2007; Bittner et al., 2007). Furthermore, childhood anxiety rates have increased dramatically since the COVID-19 pandemic, underscoring the timely importance of effective early intervention (Racine et al., 2021). Currently available interventions such as exposure-based cognitive behavioral therapy (CBT) help to resolve anxiety disorders in children as young as 4–7 years old, however, nearly half of those treated continue to experience clinically significant symptoms (Hirshfeld-Becker et al., 2010; Monga et al., 2015). To pave the way for novel, more effective treatments, the Research Domain Criteria (RDoC) advocates that symptoms of psychopathology (e.g., anxiety) be studied in relation to observable behaviors and neural circuits within constructs relevant to psychopathology (e.g., cognitive control and threat). The experimental medicine initiative takes RDoC a step further, proposing an iterative process in which brain-behavioral markers of RDoC constructs are modulated to reduce psychopathology while also elucidating mechanisms of illness to guide the further design of targeted interventions (Insel, 2014, 2015). Applying RDoC within the experimental medicine framework, we sought to test a novel, mechanism-based intervention targeting effortful control—the ability to regulate attention and behavioral impulses—to reduce anxiety in young children. This intervention, “Camp Kidpower”, uses play-based effortful control (EC) training games, delivered in a camp-like format, to boost brain- and behaviorally indexed EC in anxious preschoolers.

## Effortful control (EC) as an intervention target

Effortful control (EC) is a temperament construct that refers to a child’s ability to regulate attention and inhibit behavioral impulses (Rothbart et al., 2006). While traditional temperament literature often assumes EC to be a stable trait, we conceptualize it here as a facet of cognitive control that is highly sensitive to development, maturing progressively throughout childhood and adolescence (Atherton et al., 2020). EC has been proposed to encompass two distinct but related processes: executive control (often termed “cool” EC) and delay of gratification (“hot” EC; Kim et al., 2013; Lawler et al., 2023; Sturge-Apple et al., 2017).

“Cool” EC refers to the ability to direct attention and inhibit responses to stimuli that are neutral, decontextualized, and abstract (Sturge-Apple et al., 2017). This process is observed in tasks requiring cognitive flexibility (e.g., task-set switching), sustained attention to goal-relevant information, suppression of irrelevant information (i.e., inhibitory control), and continuous monitoring of actions (i.e., performance monitoring; Miyake et al., 2000). In contrast, “hot” EC pertains to the ability to delay gratification for hedonically appealing rewards (Mischel et al., 1988) and is conceptualized as an emotionally charged domain of EC, where tasks evoke approach motivation by presenting potential prizes or enhanced rewards (Sturge-Apple et al., 2017). Given its complex nature, EC is also referred to in developmental literature as self-regulatory control, cognitive control, or executive function (Nigg, 2017). Importantly, EC has been proposed as a transdiagnostic construct of relevance to the expression of different forms of psychopathology (Ip et al., 2019), including anxiety (Fitzgerald et al., 2021).

**Figure 1.**
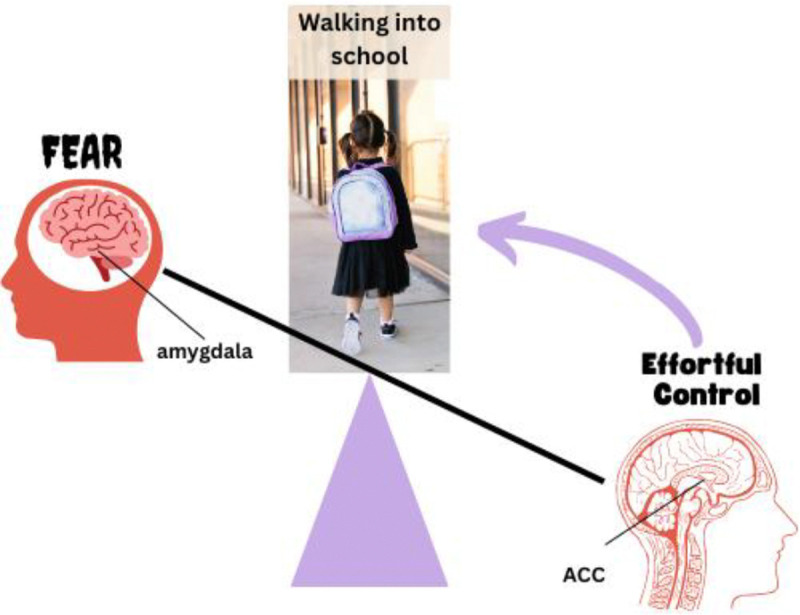
EC/Threat-reactivity Imbalance Model of Anxiety: As displayed, insufficient regulation of threat reactivity (i.e., low EC capacity; ACC) hinders the ability for children with anxiety to redirect attention away from internal worries and inhibit amygdala-driven avoidance behaviors (e.g., inhibit fear and walk into school).

On a neurobehavioral level, the dorsal anterior cingulate cortex (dACC) is thought to recruit EC through its role in performance monitoring, particularly the detection of errors to signal the need to heighten EC and thereby optimize ongoing performance to achieve task goals, whereas the dorsolateral prefrontal cortex (dlPFC) implements the specified control (Botvinick et al., 2004; Kerns et al., 2004; MacDonald et al., 2000). Neurobehavioral evidence suggests that insufficient capacity of dACC- and dlPFC-based effortful control systems relative to threat reactivity circuits (e.g., amygdala) may drive anxiety from early in life (Duval et al., 2015; Fitzgerald et al., 2021; Hur et al., 2019). In fact, given that neural systems mediating EC mature later than those underlying threat reactivity, vulnerability to anxiety disorders may be especially high in childhood (Beesdo et al., 2009). There are several possibilities as to how this imbalance causes and maintains anxiety (e.g., low EC combines with normative threat reactivity or normative EC combines with excessive threat reactivity). For example, we posit that in healthy children a mismatch between a desired behavior (e.g., walking into school on the first day of kindergarten) and feared outcome (e.g., separating from a parent) is appropriately detected as an error by the dACC. This error serves as a call to recruit adaptive control – instantiated by the dorsolateral prefrontal cortex (dlPFC) to enable appropriate, on-task behavior (i.e., walking into school). In children with anxiety, deficits in brain-behavioral mechanisms for error signaling and adaptive control may reduce capacity for on-task behavior and elevate sensitivity to “normative” levels of fear, leading to avoidance behaviors and escalating anxious distress. Alternatively, normative levels of EC capacity could be “overwhelmed” by abnormally high levels of threat reactivity to disrupt task-appropriate behavior. In either case, insufficient regulation of threat reactivity (i.e., low EC capacity) hinders the ability for children with anxiety to redirect attention away from internal worries and inhibit anxiety-driven avoidance behaviors. Thus, increasing EC capabilities should help children to overcome anxiety by countering normative-to-high levels of threat reactivity (Figure 1.).

### Error-related negativity (ERN) and time frequency interchannel phase synchrony (ICPS)

Performance monitoring by the dACC can be observed through the error-related negativity (ERN), an electrophysiological response that occurs after an error is committed. Time Frequency Interchannel Phase Synchrony (ICPS; Cohen, 2014) reflects the transmission of this error-related signal to the dlPFC to implement the specified control. The ERN is a negative deflection in the response-locked event-related potential (ERP) that occurs within 100ms following an erroneous response and is primarily localized to the dACC (Ladouceur et al., 2007). The ERN may reflect the brain’s signal for increased EC to adjust behaviors in response to errors. ERN amplitude has been found to increase with development (Davies et al., 2004; Ip, Liu, et al., 2019; Lo et al., 2017; Tamnes et al., 2013), and is associated with better behavioral performance on EC-demanding tasks, including in young children (Liu et al., 2020). ICPS measures the consistency of phase oscillations between two channels (i.e., functional connectivity) for each frequency window (e.g., theta, 4 – 8 Hz; delta, 1 – 4 Hz), at each timepoint across trials. Few studies have examined error-related ICPS in young children. Yet, an existing study of young children found age-related increase in theta-based ICPS between frontocentral and frontolateral sites after errors (Morales et al., 2022), converging with studies of adults and adolescents (Buzzell et al., 2019; Cavanagh & Frank, 2014), as well as age-related increases in delta-based ICPS between these sites across early childhood (Morales et al., 2022). These age-related enhancements of ICPS may reflect the maturation of functional connectivity between mediofrontal cortex (e.g., ACC) and dlPFC and motor regions, involved in instantiating control (Buzzell et al., 2019; Cavanagh et al., 2009).

## EC training as an intervention for anxiety in preschoolers

EC training has been previously tested as a strategy to facilitate the development of executive function in young children, but interventions implementing EC training to treat childhood anxiety are lacking. In community samples of children, EC training has been found to improve performance on EC-demanding tasks, including both “cool” and “hot” EC tasks and those not specifically trained, translating to better clinical and academic outcomes (Barnes et al., 2021; Lawler, J., 2015; McClelland et al., 2019; Muir et al., 2023). EC training has also shown promise in treating several developmental psychopathologies associated with cognitive deficits in children as young as age 3, particularly ADHD (Halperin et al., 2013; Veloso et al., 2020 for a review). In 11–14-year-olds from a community sample with elevated anxiety, working memory training has been found to increase non-trained EC function (inhibitory control) and reduce anxiety (2016). Studying the effects of EC training on brain, behavior and anxiety symptoms in even younger children leverages a developmental period when EC capacity is particularly malleable and may be most likely to improve with training (Diamond & Lee, 2011). Indeed, preliminary evidence from an open-label pilot study found increased ERN, improved EC behaviors, and reduced anxiety after a camp-like EC training intervention (Schroder et al., 2022). Collectively, this research provides strong justification for a randomized clinical trial to test EC training to reduce anxiety in preschoolers.

## The current study

To assess the degree to which increasing EC may help young children overcome anxiety, the “Camp Kidpower” study uses a randomized clinical trial design to test EC training compared to an active comparator in clinically anxious preschoolers. The EC training condition involved the titration of progressively more difficult game-like exercises that target selective attention, inhibitory control, set shifting and working memory. An active comparator, child-led play (CLP), controlled for non-specific effects of attending camp (e.g., exposure to separation from parents, novel environment, unfamiliar children and camp counselors). Both EC training and CLP were delivered to small groups of anxious children in five, weekly 3-hour sessions with daily at-home practice of games encouraged between camps. Brain and behavioral markers of EC, including non-trained EC behaviors and neurophysiological indices (i.e., ERN and ICPS), and anxiety were measured before and after both interventions to test whether treatment with EC training, relative to CLP, increases ERN and/or ICPS, improves performance on non-trained EC tasks, and reduces anxiety in preschoolers. Additionally, data were collected to assess whether increases in neurophysiological and behavioral measures of EC relate to reductions in clinically significant anxiety. Finally, for both the EC and CLP camp conditions, exploratory analyses were planned to test dosing effects of intervention efficacy, with “dose” measured as frequency and duration of at-home practice and number of intervention sessions attended.

## Methods

### Recruitment

Participating families were recruited at two sites: Michigan State University in East Lansing, MI, and Columbia University/New York State Psychiatric Institute in New York, NY. Participants were recruited using Institutional Review Board (IRB)-approved flyers and advertisements that are distributed throughout the community and posted on websites such as Facebook and Twitter. The study teams also received referrals from university-based and community-based daycares, anxiety clinics, and parent support groups. Written informed consent was obtained by a study coordinator from a parent/legal guardian both for themselves and for their child. All participants were advised that research is entirely voluntary, they may withdraw participation at any time, or they may be withdrawn by the study team if the intervention was no longer appropriate or causing worsening of symptoms. It was also requested that, should the parent consider additional treatment for their child, they discuss this with the study team first, and may need to be withdrawn from the study as a result. Families were compensated for participation.

### Condition randomization

Families were randomly assigned to either the EC camp or CLP camp condition using single-blinded block randomization guided by a study statistician. In accordance with NIMH guidance on individually randomized group-treatment (IRGT) trials, cohorts of 8–12 participants were recruited, consented and confirmed as eligible prior to randomization into either EC or CLP conditions. At the time of randomization, study coordinators were notified by the study statistician which camp condition each participant was to receive; this information remained masked from study clinical assessors, who were deliberately omitted from all camp-related meetings and communications. If necessary, unblinding of participants was possible by contacting the study coordinator. Each camp condition then comprised 4–6 children, a decision based on pilot work showing that the EC camp was feasible and well-received when delivered to similar sized groups. Additionally, groups were randomly sequenced to ensure the same number of groups for each treatment condition. Conditions were either delivered back-to-back (e.g., one condition during early fall, followed by the other condition in late fall) or on concurrent dates (e.g., one condition on Saturday mornings, followed by the other condition on the same Saturday afternoons), depending on time of year and staff availability.

### Participants

#### Child Participants: Inclusion/Exclusion criteria

Enrollment of 90 child subjects occurred over 3 years. Inclusion criteria required that child participants be between the ages of 4.0–5.99 years at the time of informed consent and meet diagnostic criteria for an anxiety disorder as assessed by a trained clinician via clinical interview (Anxiety Disorder Interview Schedule-Parent Version; ADIS-P). One of the following was required to be primary or co-primary: social anxiety disorder, separation anxiety disorder, generalized anxiety disorder, panic disorder, and/or obsessive-compulsive disorder. Of note, primary specific phobia and/or selective mutism alone were not sufficient for study inclusion. A diagnosis of specific phobia does cause impairment daily, rendering assessment of intervention-related change effects unfeasible. Similarly, selective mutism alone differs significantly from the other “core” anxiety disorders, and thus not is directly comparable when assessing symptom severity and treatment effects. Child participants were required to be fluent in English and needed written informed consent by a parent to participate. Child participants were not eligible if they presented with a history of: head injury, serious medical or neurological illness, major depressive disorder, post-traumatic stress disorder, neurodevelopmental delay, autism spectrum disorder (ASD), intellectual disability, or a recent history of physically aggressive behaviors that caused harm to other children. Child participants were excluded from the study if they were currently taking medications that affect the central nervous system or were receiving psychotherapy or other behavioral interventions; had a history of aggressive behaviors towards other children; or had siblings who were currently (or previously) enrolled.

#### Parent Participants: Inclusion/Exclusion criteria

Parent participants were required to be living with the child participant at least 50% of the time to provide accurate report on child anxiety symptoms before, during and after camp. Parent participants were required to be fluent in English and have access to the internet and a webcam to complete remote study components. Additionally, parent participants needed to provide written informed consent for both themselves and their child for both parties to participate. Parent participants were not eligible to participate if they were under 18 years of age or were unable to meet the scheduling demands of the study.

### Intervention

Note: See **Appendix A** for descriptions of all activities per EC camp sessions, **Appendix B** for descriptions of all activities per CLP camp session, and **Appendix C** for game details and camp schedules per both conditions.

To isolate the effect of EC training on outcome measures, both Camp Kidpower conditions (EC and CLP camps) were delivered using the same structure. Each camp involved five ~3-hour sessions administered over 4 weeks (one weeknight and four Saturdays) including 4–6 children paired 1:1 with a junior “camp counselor.” Junior camp counselors included senior level high school students, college undergraduates, and first year master’s students with a range of clinical experience (e.g., many knew they enjoyed working with young children but had no direct clinical experience). A senior or “lead’ camp counselor with clinical experience (e.g., senior level clinical psychology graduate student or higher education) supervised intervention delivery by junior counselors to child participants. Junior and lead camp counselors were trained in the interventions over two sessions prior to working with child campers. Both conditions began with a welcome activity followed by either EC training games or child-led play. After a brief break and snack, children continued EC games or CLP followed by a final group activity. During the last 30 minutes of camp, parents were taught how to play camp games (either EC training or CLP, depending on condition) by children and counselors. Camp concluded with the assignment of camp games as homework and encouragement to play games 15 min per day between camp sessions.

#### Effortful Control Condition (EC camp)

Building from cognitive training, early childhood intervention, and anxiety treatment literature (Diamond et al., 2007; Espinet et al., 2013; J. M. Lawler et al., 2019; Rueda et al., 2005; Thorell et al., 2009), the EC camp used short game-like exercises adapted from Halperin and colleagues’ published work with ADHD that emphasizes teaching selective attention, response inhibition, set shifting skills, and working memory skills (Halperin et al., 2020). Game difficulty was gradually increased within and across camp sessions. Importantly, the EC camp targeted multiple elements of EC/EF, titrated game difficulty, incorporated physical activity, and encouraged social bonding, all based on prior work showing that these key elements characterize training interventions that are most likely to yield transfer to non-trained behaviors (Diamond & Lee, 2011). To ensure that children could complete games during camp, counselors worked 1:1 with campers, helping each child identify and consider mistakes in the context of game rules by reciting those rules aloud during play to “self-coach” towards correct performance. This approach is known as “reflection training” and has been found to help children to persist through challenges and improve training outcomes in prior work (Espinet et al., 2013). Success in playing games was further supported by “group think,” during which children shared strategies with one another for performing the EC camp games. Finally, all games could be played at different levels of difficulty, making it possible to reduce or increase difficulty as needed to ensure that each child was able to complete games successfully while still being challenged. See **Appendix A** for descriptions of all games, including difficulty levels and reflection training examples.

One example of an EC camp exercise is *Red Light, Green Light*. In this game, children stand on a starting line and work their way towards an experimenter who is on the finish line. When the camp counselor gives the “Green Light” visual and auditory cues, children must move towards the finish line. When the experimenter gives the “Red Light” visual and auditory cues, children must stop and freeze. As shown in **Appendix A**, difficulty can be increased in Red Light, Green Light in multiple ways. One way is through manipulating the “Green Light” and “Red Light” cues, starting with the camp counselor holding up green and red visual cues and eventually transitioning to the experimenter only giving verbal cues. Additionally, the camp counselor can switch the rules such that the “Green Light” signal means stop and the “Red Light” signal means go, as well as incorporate trick cues with no meaning (e.g., “Yellow Light”). As illustrated, this game involves multiple EC/EF skills, including selective attention, inhibitory control, set shifting and working memory as children must selectively attend to and remember specific rules of the game while practicing their shifting and inhibitory control skills.

During reflection Training in Red Light, Green Light (see **Appendix A**), the camp counselor can ask the child to verbalize the rules of the game (e.g., “What does ‘Red Light’ mean? What does ‘Green Light’ mean?”). The counselor can also brainstorm strategies with the child to help them overcome their errors as game difficulty is increased (e.g., “Freezing when you’re running so fast can be hard! What do you think is a good way to hold still when we want to go fast? Right, let’s try slowing down!”). To ensure that the child is actively reflecting during game exercises, throughout the game, the camp counselor can also check in with the child, asking them to identify and correct errors as they play (e.g., “[Counselor] said ‘Red Light’, but I kept going. What should I do when I hear ‘Red Light’?”).

#### Behavioral management techniques

Informed by our pilot study, we incorporated behavioral management techniques to motivate child and group participation and help children stay on-task. Behavioral management techniques were introduced as “camp rules” during circle time at the beginning of each session. One example of a successful technique was the “elephant ears” gesture, during which the lead camp counselor reminded children that when a counselor places their hands by their ears, it meant to listen carefully. Another technique involved reinforcing positive behaviors through stickers and “power stars” (multi-colored paper star cutouts). If a child followed instructions and engaged in a particularly challenging camp task, a camp counselor rewarded the child with an individual sticker. Once that child collected 4 stickers, they added a “power star” to the collective group jar. Each child had the opportunity to earn 1 power star per camp game (4 games total), and children were told that if they collected enough power stars as a group by the end of the camp session, they would be rewarded with a special activity (i.e., playing with parachute for 10 minutes, each choosing a prize, etc.). Given that behavioral modification may influence EC/EF skills, the above techniques were only utilized during the EC camp condition (not the CLP camp).

##### Child-Led Play Condition (CLP camp):

CLP is a type of special one-on-one “playtime” where the child directs and leads the interaction. CLP is commonly included as a component of other evidence-based treatments for preschool-aged children, such as parent-child interaction therapy (PCIT), in order to improve parent-child attachment and externalizing behaviors such as aggressive behaviors (Woodfield & Cartwright, 2020). In the current study, the CLP camp permitted identification of specific effects of the EC training by controlling for confounds including a) participation in a fun playgroup; b) interaction with new children during activities; c) separation from parents; and d) time spent with new adults. CLP “playtime” included structured play activities such as art, dramatic play, and story time around a specific theme (e.g., nature, music, etc.), but no EC games nor reflection training.

#### At-home practice

Across conditions, parents were asked to complete “homework” at home for 20 minutes daily between camp sessions. To facilitate homework completion, parents were required to attend: 1) an introductory parent meeting before camp begins explaining the Camp Kidpower condition (EC vs. CLP) and homework expectations; 2) a check-in parent meeting in the middle of camp to answer questions about how homework was going; 3) the last ~30 minutes of each camp session to receive a report of that session’s activities and specific homework instructions. Additionally, parents tracked time spent on homework via a daily Qualtrics survey (see **Appendix D** for homework details) that was monitored daily by staff. If challenges in completing homework occurred, staff called parents to help problem solve over the phone. Additionally, parents met after the second camp session to debrief with one another and troubleshoot, guided by the lead camp counselor. Parent reports on homework activities were collected to enable exploratory analyses to test potential moderating effect of dose (homework frequency, duration, enjoyment) on multi-level outcome measures.

#### EC camp homework

EC Camp homework involved practicing two games that were learned during camp. At the end of each camp session, two individualized games were assigned by the child’s camp counselor: one that was easy and fun for the child, and one that proved more challenging. Parents were provided instructions for the two games, including how to titrate game difficulty and how to incorporate reflection training. Parents of children enrolled in the EC camp were also asked whether they used reflection training.

#### CLP camp homework

CLP Camp homework involved practicing child-led play at home. At the end of each camp session, parents were provided with an overview of the daily camp theme and exercises (e.g., art activity, Storytime book), as well as a suggested at-home activity in line with that week’s session (see **Appendix B**). Parents were provided instructions on how to engage in child-led play at home, including tips on how to imitate their child’s movements, describe play actions, and praise appropriate behaviors. The lead camp counselor was made available at the end of each camp session to answer any parent questions that arose during at-home practice.

### Pre- and post- intervention assessment

All assessments were conducted at lab visits within ~2 weeks of intervention start and end dates. Table 1 provides a summary of all pre- and post- intervention assessment measures.

#### Neurophysiological measures of EC

Neurophysiological (EEG) data were collected by trained technicians. To assess neurophysiological indices of EC, we used the “Zoo Task,” a child-friendly Go/No-Go paradigm that has been found to reliably elicit ERN in preschoolers (Grammer et al., 2014). Before performing the task, children were instructed: “Animals have escaped from the zoo! Can you help the zookeeper and her orangutan helpers put them back in their cages?”. Children then viewed a series of animals on a computer screen and were asked to press a button when a new animal appeared (Go trials), unless it was an orangutan (i.e., withhold pressing button, NoGo trials). The task included 8 blocks, each containing 30 unique animals (Go trials) and 10 orangutans (NoGo trials) in random order.

Strategies to optimize ERP data collection in clinically anxious preschoolers have been developed in our lab during pilot testing and are described elsewhere (see Horbatch et al., 2021 for details). Briefly, these strategies include: 1) Email and telephone contacts with parents prior to the initial visit to model language that can be used to explain EEG to children. 2) A “social story” e-booklet outlining step-by-step details of the visit guided by “Mr. Bananas” (monkey stuffed animal) to elicit child questions about EEG cap/sensors, and 3) A “prep visit” to the EEG space, prior to data collection, to allow the child to learn about EEG equipment, build rapport with technicians, and practice setup on a doll head. Children sit in a comfortable chair; while playing the Zoo task, an electroencephalogram (EEG) will be recorded from 32 Ag/AgCl scalp electrodes and two mastoid electrodes using the BioSemi Active 2 recording system. A detailed description of EEG data acquisition and preprocessing can be found in published protocols (Ip, Liu, et al., 2019; J. M. Lawler et al., 2021; Schroder et al., 2022).

#### Error-related negativity (ERN)

The ERN is defined as the average ERP amplitude in the 0–50 ms post-response window on false alarm trials; the correct- response negativity (CRN) is defined in the same window on correct Go trials. Response-locked data was baseline-corrected using a pre-response baseline of −200 to −100ms (Grammer et al., 2014). ERN amplitude was measured at electrode sites Fz, FCz, and Cz. Differentiation between these trial types will be the primary ERN measure (delta ERN) at analysis, but we will also consider ERN on error trials, as well as ERPs on correct trials (CRN) separately. Previous research indicates that the ERN demonstrates good psychometric properties across young children (Ip, Liu, et al., 2019; Morales et al., 2022) adolescents and adults (Meyer et al., 2012; Weinberg & Hajcak, 2011). The number of NoGo errors and response times (RTs) to Go trials will also be considered in analyses, as performance can affect ERN amplitude (Gehring et al., 2011).

#### Time frequency interchannel phase synchrony (ICPS)

Using the same approach previously employed by our group (Aviyente et al., 2017), complex time-varying energy time-frequency distributions (TFDs) of all the EEG signals at each channel extracted from the Zoo task (described above) were obtained using the reduced interference distribution (RID) Rihaczek distribution (Aviyente et al., 2011). RID-based TFDs offer improved time-frequency support relative to wavelets. Functional connectivity was assessed using interchannel phase synchrony (ICPS), based on the phase locking value (PLV; Lachaux et al., 1999), derived from the RID TFDs. Current source density (CSD) transform will be applied to EEG data before phase synchrony analysis to reduce volume conduction, using published methods (Tenke & Kayser, 2012). We will focus on ICPS within the theta band in the ERN time window. Consistent with previous work (Aviyente et al., 2017; Cavanagh et al., 2009), the current study will report ICPS between FCz and lateral frontal sites – i.e., medial-lateral functional connectivity – indexing control processing.

#### Behavioral measures of EC

Behavioral assessments of EC occurred before and after the intervention using a series of well-validated lab-based tasks in conjunction with computerized tasks from the NIH Toolbox Early Childhood Cognition Battery. Importantly, none of these behavioral paradigms were specifically trained through the EC camp, enabling assessment of the transfer of EC/EF skills developed during camp to non-trained tasks.

#### Computerized NIH Toolbox Early Childhood Cognition Battery

We used the computerized Flanker inhibitory control and attention task (Flanker) and Dimensional Change Card Sort (DCCS) test administered through the NIH Toolbox Early Childhood Cognition Battery on an iPad. Both the Flanker and DCCS tasks are well-validated indices of EF in 3–7-year-old children (Zelazo et al., 2013). Prior studies have suggested that both tasks have excellent developmental sensitivity across childhood, test-retest reliability, and convergent validity. For both tasks, children’s performance scores are based on a two-vector method of considering both accuracy and reaction time, and standardized scores are calculated based on established age, gender, and race/ethnicity norms.

#### Lab-based measures

##### Head-Toes Knees-Shoulders Task (HTKS; McClelland et al., 2014).

Experimenter gives child prompts such as “touch your head” and, depending on instruction context, child must either follow the command or substitute a different body part (e.g., toes instead of head). HTKS includes 20 trials, each scored as 0 = incorrect response, 1 = self-corrected error, or 2 = correct response. Total HTKS scores will be calculated by averaging all trials together, with higher scores reflecting greater overall EF skills.

##### Day/Night Stroop Task (Kochanska et al., 1996).

A measure of inhibitory control that has two parts, a control phase in which children are shown a picture of the sun and asked to say “day” or a picture of the moon and asked to say “night”; and a conflict phase in which they are asked to respond “night” when they see the sun and “day” when they see the moon. Each phase has 10 trials (after 2 practice trials), and children’s accuracy and total time to complete each phase will be recorded. Higher scores indicate better inhibitory control.

##### Dinky Toys Task (Kochanska et al., 1996).

Children are asked to view a plastic box filled with toys and tell the experimenter which toy he or she wants while holding their hands immobile in their lap for up to 2 minutes. Scores for the Dinky Toys task range from 0 (hands never left lap) to 5 (grabbed toy(s)), with lower scores reflecting greater inhibitory control. The Dinky Toys task will be administered three times at each visit (pre-intervention visit and post-intervention visit), and scores will be averaged to compute a total score.

##### Tongue Task (Kochanska et al., 1996).

Child must hold M&M on tongue without eating it (2 trials, delays of 10 and 40 seconds); scoring reflects the length of delays (in seconds) before the child ate the candy.

#### Gift Wrap Task (Kochanska et al., 1996).

Child is instructed not to peek while experimenter wraps gift; scoring reflects a composite of frequencies of peeking and verbal references to the gift.

### Behavioral Coding of Lab Tasks

Prior work indicates strong correlations across lab-based tasks (Dyson et al., 2015). For this study, coders will be trained to reliability standards according to established coding manuals, and 20% of the data will be double-coded (Chorney et al., 2015). We will conduct an exploratory factor analysis (EFA) on these tasks to examine the factor structure of EC in young children. Prior studies found that EC may be best fit by a unitary or a multidimensional (“cool” and “hot” EC) construct across these tasks (Allan & Lonigan, 2011; Ip, Liu, et al., 2019; Zelazo et al., 2013), so a composite score based on the EFA results will be computed here.

### Global rating of EC behaviors

Global measures of EC behaviors across lab-based behavioral tests at both pre- and post-intervention lab visits and during each of the five camp sessions were collected using the Preschool Self-Regulatory Assessment-Assessor report (PSRA). The PSRA is a validated 28-item questionnaire on which trained raters provide scores using a Likert scale (0 to 3) to generate a global Attention/Impulsivity score (Smith-Donald et al., 2007). This measure has been previously validated for the assessment of EC behaviors in both laboratory and “real world” settings (e.g., classroom; Bassett et al., 2012). Of note, the PSRA is adapted for optimal relevance to this study and ease of administration (see **Appendix E** for adapted PSRA). In the current study, PSRA was completed at each camp session. Student camp counselors (assigned one-on-one to the same child “camp buddy” each week) scored the PSRA for their respective camp buddies after each session. The Lead camp counselor (master’s level clinician) scored the PSRA independently for each child, then met with each student counselor to reach clinical consensus for each child after each session.

### Parent report of EC

To ensure that measurement of EC captures child behaviors across different contexts, we administered the well-validated temperament questionnaire Child Behavior Questionnaire (CBQ; Rothbart et al., 2001) to assess effortful control reported by parents, which is more likely to reflect child’s inhibitory control skills in the home and family environment.

### Anxiety symptom assessment

#### Clinician rating

Trained clinicians (master’s degree or higher) experienced in the assessment and treatment of childhood anxiety disorders delivered clinical assessments to parents. Importantly, clinical assessors were blinded to the Camp Kidpower condition into which child participants were randomized. Fidelity between clinician ratings was assessed quarterly, led by a child psychiatrist with expertise in pediatric anxiety disorders.

##### The Anxiety Disorder Interview Schedule-Parent Version (ADIS-P).

The ADIS-P was administered at baseline assessment and following completion of the study intervention. The ADIS-P is a validated, clinical semi-structured interview conducted with parents to ascertain DSM-IV diagnoses in children (Edwards et al., 2010; Kennedy et al., 2009; Lyneham et al., 2007; Rapee et al., 2005; Vreeke et al., 2013). The initial ADIS-P interview was also used to establish anxiety severity, identify comorbidities, and determine eligibility. Importantly, the rater was blinded to which intervention (i.e., EC camp vs. CLP camp) families received. For each diagnosis, the ADIS-P requires interviewers to assign a clinician severity rating (CSR) that indicates the degree of distress and impairment associated with the disorder from 0 (none) to 8 (very severely disturbing/disabling). The CSR for the anxiety disorder rated most severe at study entry served as a secondary measure of anxiety severity change (i.e., from pre- to post-intervention). The ADIS-P also generates a CSR for ADHD symptoms to be considered in secondary analyses.

##### Clinical Global Impressions-Severity and Improvement Scales (CGI-S and CGI-I ; Busner & Targum, 2007).

The CGI-S and CGI-I were used to assess anxiety severity and pre- to post-treatment improvement in anxiety. Ratings for both the CGI-S and CGI-I use a 7-point scale, from 1 “normal” to 7 “among the most severely ill” for CGI-S and 1 (“very much improved”) to 7 (“very much worse”) for CGI-I. Consistent with prior work in clinically anxious preschoolers, the CGI-S will serve as the primary outcome measure in analyses (Bilek & Ehrenreich-May, 2012; Hirshfeld-Becker et al., 2010; Monga et al., 2015).

#### Parent/teacher report

##### Spence Preschool Anxiety Scale (PAS; Spence et al., 2001).

Parent report was provided on the PAS, a validated instrument for the measurement of anxiety symptoms in young children. PAS scores were collected at both pre and post intervention visits and on a weekly basis at each camp session, providing a secondary measure of anxiety.

##### Child Behavior Checklist (CBCL).

Parents provided reports on the CBCL ages 1.5– 5 at the baseline visit (Achenbach, 2009). The CBCL quantifies externalizing and internalizing problems. The DSM-Anxiety and Attention Problems subscales were used to screen for potentially eligible subjects prior to study entry (see above). In addition, T-scores for DSM-oriented depression, anxiety and externalizing subscales (normed on child age and gender) may be used in secondary analyses.

##### Caregiver-Teacher Report Form (C-TRF; Achenbach and Rescorla, 2000).

Daycare providers or teachers provided ratings at baseline on 99 items to assess social, emotional, and behavioral functioning in children. Six syndromes have been derived from C-TRF ratings of preschoolers which may be used in secondary analyses: Emotionally Reactive, Anxious/Depressed, Somatic Complaints, Withdrawn, Attention Problems, and Aggressive Behavior (Achenbach and Rescorla, 2000).

### Threat Sensitivity

#### Behavioral measures

**The Potential Threat Task** (McGinnis et al., 2019) is a lab-observed behavioral task designed to elicit uncertainty during approach to a hidden, but benign object (e.g., a tissue box covered with a blanket). During the task, the experimenter leads the child through a dimly lit room, giving scripted statements to build anticipation such as “I have something in here to show you,” or “let’s be quiet so that it doesn’t wake up.” Approach towards the hidden object is measured with a wearable motion sensor belt to quantify avoidance – a behavioral correlate of internalizing psychopathology (McGinnis et al., 2018; McGinnis et al., 2018, 2019) that we will compare pre- and post-intervention.

**The Speech Task,** (McGinnis et al., 2019), is an adapted version of the Trier Social Stress Task for children (TSST-C; Allen et al., 2017), which has been shown to induce anxiety in children. During this task, the experimenter instructs the child in a serious tone to prepare and give a three-minute speech. The child is told that they will be judged based on how interesting their story is. A buzzer is used to interrupt the child’s story with 90 and 30 seconds remaining in the task. At each interruption, the experimenter informs the child of the time remaining in the task using a standardized script. The same motion sensor belt from the potential threat task is worn during the speech task to quantify movement patterns. Additionally, audio is recorded to be used in analysis of speech patterns indicative of internalizing disorders (McGinnis et al., 2018, 2019).

#### Parent report

Parent ratings on the Child Behavior Questionnaire-Fear scale (CBQ-Fear; Rothbart et al., 2001) and Behavioral Inhibition Questionnaire (BIQ; Bishop et al., 2003) will serve as indices of threat sensitivity. Both are well-validated questionnaires to quantify behaviorally inhibited temperament, which has been consistently demonstrated to predict anxiety disorders in children (Vreeke et al., 2013).

#### Child report

Child ratings on the Child Error Sensitivity Index (ESI; Chong & Meyer, 2019) were collected pre-intervention and post-intervention. The ESI is a 9-item questionnaire intended to measure children’s reactions to making mistakes. Research staff used hand gestures (e.g., thumbs up, thumbs down) to assist children in choosing if the prompts were: 1 = “not at all like me,” 2 = “somewhat like me,” or 3 = “a lot like me.” The Child ESI has demonstrated good internal reliability in previous work (Chong & Meyer, 2019).

### Parent psychopathology

Parent psychopathology was measured by parent self-ratings of anxiety and depression symptoms on validated measures (see Table 1 for specific measures). These ratings will be used as covariates in our analyses, as parental psychopathology may bias ratings of their children (Renouf & Kovacs, 1994) and impact child response to treatment (Hudson et al., 2015).

## ANALYSIS PLAN

### Statistical analyses

#### Analysis of data/study outcomes

To examine whether the EC camp modulates neural and behavioral indexes of EC, analyses will use a multilevel regression approach in which the child’s outcome at post-treatment (i.e., ERN, ICPS, behavioral indices of EC including NIH Toolbox and Observed EC composite scores) is predicted by the intervention condition (EC camp vs. CLP camp) while controlling for the child’s baseline score for that outcome. To assess the relationship between change in neurobehavioral markers of EC and symptoms of anxiety, we will conduct two sets of multilevel regression models for each EC mediator (i.e., ERN, ICPS, and behavioral EC). In the first set of models, post-treatment EC will be predicted as a function of the intervention condition (coefficient “a”) while controlling for baseline EC. In the second model, post-treatment anxiety (CGI-S) will be predicted as a function of the intervention and post-treatment EC score (coefficient “b”), treating baseline anxiety as a covariate.

Because the study involves five training sessions during which parent reports of weekly dose (duration and frequency of practice), parent reports of anxiety during the week (Spence PAS), and observations of EC during the weekly session (PSRA EC) are acquired, exploratory multilevel models can examine links among these variables over time for the children in the EC camp. We will use a cross-lagged model to predict current anxiety (Spence PAS) as a function of the previous week’s PSRA EC (or parent reported dose), controlling for previous week’s PAS.

Finally, to explore other potential moderators for the effectiveness of the EC camp such as parent psychopathology and child temperament, multilevel models will test whether the effects of the EC camp intervention on post-treatment are moderated by children’s levels of behavioral inhibition/fearfulness and parents’ levels of anxiety.

#### Power analyses

Of the 90 children to be randomized, our pilot data suggests that 87% will be completers with both pre and post intervention EC behavioral and anxiety measures (n = 78) and 70% with both pre and post intervention ERN and ICPS (n = 63). Effect sizes were obtained from preliminary data generated by children who received the EC camp in a pilot study. The power analyses considered the individually randomized group-treatment trial (IRGT) design based on pre- to post-intervention change scores in ERN (−6 ± 8.04), DCCS (5.71 ± 7.83), and PAS (−6.38 ± 10.74). Assuming these measures will not change for the Child-Led Play condition and assuming the same variability for the two intervention groups, the power for detecting between-group differences is estimated at 0.896, 0.944 and 0.838 for changes in neural capacity for EC (ERN, ICPS), behavioral EC, and clinician-measured anxiety (CGI-S), respectively.

## DISCUSSION

Integrating both RDoC and experimental medicine frameworks, our study aims to develop a novel, mechanism- and play-based group intervention (Camp Kidpower for Effortful Control; EC camp) to reduce anxiety in preschoolers through modulating behavioral and neural mechanisms of EC. Findings from the pilot version of this study indicated that interventions aimed at improving EC capacity may help mitigate anxiety in young children (Schroder et al., 2022). The current study protocol details a group-randomized controlled trial for anxious 4–6 year-olds, which builds upon pilot data as well as literature in cognitive training, early childhood intervention, and anxiety treatment. By incorporating an active comparator (Camp Kidpower for Child-Led Play; CLP camp) and utilizing a multilevel approach to assess outcomes, results from this study will provide vital support for the mechanistic and practical plausibility of the EC camp intervention. Furthermore, these data will inform future studies on EC relative to baseline threat reactivity in children with anxiety.

Taken together, results from this study will inform decisions regarding whether the intervention shows the potential for improving clinical outcomes (including the strength of the association between target engagement and clinical benefit).

### Implications and future directions

Our novel Kidpower intervention, aimed at enhancing EC capacity, holds four significant implications and future directions. First, conventional cognitive-behavioral therapy (CBT) faces a systemic barrier due to its high cost and limited accessibility for families, particularly in community settings (Aarons et al., 2009; Roundfield & Lang, 2017). The novel EC camp introduced in our study addresses this issue by allowing for dissemination of the treatment in a range of clinics, schools and community centers. Moreover, community members can be trained as camp counselors, thereby making treatment less costly and reducing the burden on the medical system.

Second, while the Kidpower EC camp primarily targets EC and anxiety in young children, it could also prove effective in treating children with comorbid anxiety and attention deficit/hyperactivity disorder (ADHD). This assertion is supported by research indicating that children with ADHD exhibit deficits in EC (Barkley, 1997; Murray & Kochanska, 2002), and EC training has demonstrated effectiveness in alleviating ADHD symptoms (Halperin et al., 2020). Subsequent studies could compare the treatment efficacy of EC training for children with anxiety alone versus those with comorbid anxiety and ADHD. Our study will allow for preliminary analyses of this question, as we included children with ADHD in Camp Kidpower.

It is crucial that future work investigate how children’s age and baseline EC levels may moderate treatment efficacy. Previous studies on school-based executive function interventions suggest that younger children and those with lower baseline EC levels may benefit the most from EC training (Diamond, 2012). A nuanced understanding of which children are positioned to benefit most from EC training (for example, younger cohorts or those with EC-related psychopathology such as ADHD) could allow future work to better individualize such treatment for childhood anxiety. It is possible that baseline measurement of EC may be used to predict those most likely to benefit from the treatment outlined in this paper.

Finally, future intervention study designs could benefit from a multisystem approach (i.e., beyond a sole focus on the child’s anxiety), including consideration of caregiver emotion regulation skills (Carr, 2014). Research has indicated that limited access to emotion regulation skills in caregivers may mediate the intergenerational transmission of internalizing problems, suggesting that enhancing caregivers’ emotion regulation skills could break the cycle of intergenerational transmission of risk (Ip et al., 2021). Subsequent studies could expand the EC training protocol to include a caregiver module, focusing on reducing parenting stress and enhancing emotion regulation skills.

#### Study limitations

This study has several limitations. First, we acknowledge that we only focus on anxiety disorders in a narrow age range (4.0–5.99 years), which limits the generalizability of our findings to other age groups and types of diagnoses. Second, our study aims to collect pilot RCT data, thus study conclusion should be interpreted with cautious. Third, camps are conducted throughout the school year and summer, which may present seasonal effects; future studies with longer intervention duration would benefit from examining the effects of timing of the intervention. Fourth, study marketing materials and consent forms indicate that the camp is designed to lower anxiety, so there is potential for social desirability bias for parents to rate lower anxiety at the follow-up visit, although clinician ratings of anxiety – our primary outcome measure – will be blinded to which intervention (i.e., EC camp vs. CLP camp) families receive. Hence, this may reduce reporting bias. Fifth, because the Kidpower Study is currently only offered in English, our intervention is currently restricted to English-speaking families. To examine the generalizability of our intervention and to reach a wider range of diverse families, we recommend future iterations of the study protocol to translate all research/intervention procedures and materials to other languages (e.g., Spanish). Finally, the time- and effort-intensive nature of this study for families with young children may compromise the ability for participants to commit and adhere to the full study protocol. Future iterations of the study should aim to over-recruit to account for a high expected number of families who become lost to follow-up or withdraw before completing all study procedures.

#### Ethics

Study procedures are approved by the New York State Psychiatric Institute (NYSPI) Institutional Review Board (IRB). Risks associated with participation in the study are considered minimal and include mild psychological distress and discomfort when responding to interview questions or completing questionnaires, potential for loss of privacy, minor physical discomfort while completing the EEG, and risk of exposure to COVID-19 while traveling to/attending in-person visits. To protect confidentiality, personal identifiers (e.g., names, contact information) are stored separately from the study record for each participant. Information linking indirect identifiers with participants’ personal information is stored in a password-protected file on a secure server. The principal investigators will monitor patients closely throughout their participation in the study and report all important protocol modifications, unexpected or adverse outcomes, and required interim analyses to relevant parties (e.g., other investigators, IRB, funding agency, sponsor). Additionally, an independent data safety monitoring board (DSMB) will assess data entry, security, and safety practices yearly. Audits of this treatment protocol will be conducted on a regular basis by the sponsoring institution’s IRB, who may make decision to terminate the trial.

#### Trial Status

This protocol is presented as version 1, dated June 25, 2024. Recruitment began on July 1, 2021, and was completed on March 1, 2024. Although recruitment has been concluded, the protocol submission was delayed due to funding limitations and staff changes.

#### Dissemination policy

Scientifc papers and professional presentations of the study results are expected to be disseminated for scientists, mental health providers, parents, and teachers. Each author’s contribution will be disclosed upon dissemination. Writing of study findings will be conducted by those who collaborated in the implementation and data collection of the study. In addition, co-authorship for publications will be made available to other potentially interested researchers.

## Supplementary Material

Supplementary Files

This is a list of supplementary files associated with this preprint. Click to download.

• KPProtocolPaperAppendix6.25.25.docx

• SPIRITchecklistProtocolPaper6.10.25.docx

## Figures and Tables

**Table 1.** Multi-Level Outcome Measures - Game / Survey descriptions

Level of Analysis Construct	Measure	Modality	Administration	Personnel	Timepoint	Description
**Brain**						
Cognitive Control	ERN	EEG	E-prime	RA	Pre- and post-camp	The ERN is a negative deflection of the EEG signal from midline frontal electrode follow an error by ~0–150 msec). To derive the ERN, EEG data was collected while children performed was elicited by errors of commission on the child-friendly Go/No-go paradigm, the Zoo task. Errors were defined as “false alarms” on No Go trials.
	ICPS	EEG	E-prime	RA	Pre- and post-camp	ICPS measures the consistency of phase oscillations between two channels (i.e., functional connectivity) for each frequency window (e.g., theta, 4 – 8 Hz; delta, 1 – 4 Hz), at each timepoint across trials.
**Behavioral**						
Executive Control (“Cool EC)	Zoo task	Computer test	E-prime	RA	Pre- and post-camp	Child is instructed to click for all animals except orangutans (child-friendly Go/No-go paradigm)
	NIH Toolbox: Dimensional Change Card Sort	Computer test	iPad	RA	Pre- and post-camp	Child sorts cards by one feature (color), then another (shape).
	NIH Toolbox: Flanker Task	Computer test	iPad	RA	Pre- and post-camp	Child is required to focus on a given stimulus while inhibiting attention to stimuli flanking it.
Reward (“hot” EC)	Head-Toes-Knees-Shoulders	Observed behavior	In-person behavioral observation	RA	Pre- and post-camp	Children must complete the opposite of the experimenter’s instruction, with the task becoming progressively harder across trials.
	Day/Night Stroop Task	Lab observed behavioral tasks	In-person behavioral observation	RA	Pre- and post-camp	Child provides incongruent responses to different picture stimuli.
	Dinky Toys	Lab observed behavioral tasks	In-person behavioral observation	RA	Pre- and post-camp	Child must verbalize their desired toy to experiment without touching or grabbing the toy.
	Tongue Task	Lab observed behavioral tasks	In-person behavioral observation	RA	Pre- and post-camp	Child must hold an M&M on tongue without eating for a set period.
	Gift Wrap	Lab observed behavioral tasks	In-person behavioral observation	RA	Pre- and post-camp	Child is instructed not to peek while the experimenter wraps a gift.
Threat Sensitivity	Potential Threat Task	Lab observed behavioral tasks	In-person behavioral observation with motion sensor	RA	Pre- and post-camp	Child approaches a potentially threatening, unknown hidden object in a dimly-lit room.
Threat Sensitivity	Speech Task	Lab observed behavioral tasks	In-person behavioral observation with motion sensor	RA	Post-camp only	Child is instructed to tell a 3 min. impromptu story while told they are being videotaped
Behavioral Regulation	PSRA	Camp counselor-administered	Paper questionnaire	Lead Camp Counselor	Pre-camp, 1x after each camp session, and post-camp	The Preschool Self-Regulation Assessment-Assessor Report is designed to assess self-regulation in emotional, attentional, and behavioral domains through a global report of children’s behavior.
**Temperament**						
Effortful Control	CBQ	Adult report on child	Online questionnaire	Parent	Pre-camp only	The Children’s Behavior Questionnaire is designed to provide a detailed assessment of temperament in children 3 to 7 years of age.
	BRIEF-P	Adult report on child	Online questionnaire	Parent	Pre- and post-camp	The Behavior Rating Inventory of Executive Function-Preschool Version is a standardized questionnaire that measures executive functioning in preschoolers.
	BIQ	Adult report on child	Online questionnaire	Parent	Pre- and post-camp	The Behavioral Inhibition Questionnaire assesses behavioral inhibition six contexts, subdivided into two domains: social novelty (relative to unknown adults, peers, and performance in front of others) and situational novelty (relative to unknown situations, separation, and physical challenges).
**Clinical Assessment**						
Diagnosis and severity	ADIS	Clinician-administered	Remote (i.e., Zoom) assessment	Clinician	Pre- and post-camp	The Anxiety Disorders Interview Schedule is designed to diagnose anxiety and other disorders. It provides categorical diagnoses and severity ratings for each.
Functional impairment	CGI	Clinician-administered	Remote (i.e., Zoom) assessment	Clinician	Pre- and post-camp	The Clinical Global Impressions scale is designed for the clinician to quantify and track patient progress and treatment response over time.
Anxiety severity	Spence PAS	Adult report on child	Online questionnaire	Parent	Pre-camp, 1x after each camp session, and Post-camp	The Spence Preschool Anxiety Scale assesses a wide range of anxiety symptoms in preschool-aged children.
Anger regulation	MAP-DB	Adult report on child	Online questionnaire	Parent	Pre-camp, post-camp, and at 6–12-month follow-up	The Multidimensional Assessment of Preschool Disruptive Behavior (MAP-DB) assesses temper loss in terms of tantrum features and anger regulation. The four MAP-DB dimensions are: Temper Loss, Noncompliance, Aggression, and Low Concern for Others.
Broad band psychopathology	CBCL-Parent	Adult report on child	Online questionnaire	Parent	Pre-camp and at 6–12-month follow-up	The Child Behavior Checklist is a component of the Achenbach System of Empirically Based Assessment, used to detect behavioral and emotional problems in children and adolescents.
	C-TRF	Adult report on child	Paper questionnaire	Teacher	Pre-camp only	The Child Teacher Report Form allows teachers to rate the child’s academic performance and adaptive functioning, the appropriateness of the child’s behavior, how much the child is learning, how hard the child is working, and how happy the child appears.
**Parent Psychopathology**						
Anxiety	BAI	Adult report on self	Online questionnaire	Parent	Pre- and post-camp	The Beck Anxiety Inventory (BAI) is a 21-question inventory used to assess symptoms and severity of anxiety in individuals ages 17 and older.
Worry	PSWQ	Adult report on self	Online questionnaire	Parent	Pre- and post-camp	The Penn State Worry Questionnaire (PSWQ) The PSWQ is a 16-item inventory designed to measure the trait of worry in adults. The scale measures the excessiveness, generality, and uncontrollable dimensions of worry.
Depression	PHQ-9	Adult report on self	Online questionnaire	Parent	Pre- and post-camp	The PHQ-9 is the 9-item depression module from the full Patient Health Questionnaire (PHQ). The instrument is used to detect depression symptoms and severity in adults.
	ATQ	Adult report on self	Online questionnaire	Parent	Pre- and post-camp	The Automatic Thoughts Questionnaire (ATQ) is a 30-item scale intended to measure the frequency of automatic negative thoughts (negative self-statements) associated with depression in adults.
Parenting Style/Stress	PSI-4	Adult report on self	Online questionnaire	Parent	Pre- and post-camp	The Parenting Stress Index (PSI) is designed to identify at-risk or problem areas in the child’s or parent’s behavior. The PSI-4 focuses on three major domains of stress, including child characteristics, parent characteristics, and situational/demographic life stress.
	CCNES	Adult report on self	Online questionnaire	Parent	Pre- and post-camp	The Coping with Children’s Negative Emotions Scale (CCNES) presents 12 hypothetical scenarios in which a child or adolescent gets upset or angry. Parents are asked to identify how they would respond to each scenario. The CCNES involves six subscales, including emotion focused, problem-focused, minimization, punitive, expressive encouragement, and distress responses.
Emotion Regulation	DERS	Adult report on self	Online questionnaire	Parent	Pre- and post-camp	The Difficulties in Emotion Regulation Scale (DERS) measures emotion regulation problems. The 36 items self-report scale asks parents how they relate to their emotions. The DERS involves six subscales, including nonacceptance of emotional responses, difficulty engaging in goal-directed behavior, impulse control difficulties, lack of emotional awareness, limited access to emotion regulation strategies, and lack of emotional clarity.
Other						
Error Sensitivity	ESI	Child report on self	In-person verbal report	Child (assisted by RA and parent)	Pre- and post-camp	The Error Sensitivity Index is a 9-item questionnaire designed to measure children’s fear of making mistakes or adverse reactions to making mistakes.
	CBQ	Adult report on child	Online questionnaire	Parent	Pre-camp only	The Children’s Behavior Questionnaire is designed to provide a detailed assessment of temperament in children 3 to 7 years of age.

**3.3. Data collection and Measures.** All tasks will be counterbalanced to prevent order effects.

Figure 3.1. Overview of study measures.					
RDoC Domain	RDoC Subdomain	RDoC Unit	Task Title	Time	Measurement Unit
*NP Set Up*	---	---	---	*25min*	---
Negative Valence	Acute Threat/Fear	Neurophysiology	Video Clips	*12min*	Fear Potentiated Startle
Positive Valence	Response to Reward	Neurophysiology	Guessing Game	*15min*	Feedback Related Negativity
*NP Take Down*	---	---	---	*15min*	---
*Regulatory Period*	---	---	*Free Play*	*5min*	---
Negative Valence	Acute Threat/Fear	Behavior	Spider	*3min*	Avoidance and Startle
Positive Valence	Response to Reward	Behavior	Bubbles	*5min*	Positive Affect

**Table 2.** Participant Timeline: Schedule of enrollment, interventions, and assessments

	Baseline Visit	Camp Sessions					Post-Camp Visit	6–12 mo Follow-Up
		1	2	3	4	5		
**STUDY-TEAM ADMINISTERED**								
**Informed Consent**	x							
**ADIS-P**	x						x	
**CGI (S/I)**	x						x	
**PSRA**	x	x	x	x	x	x	x	
**PARENT (ON CHILD)**								
**Demographics**	x							
**ASQ**	x							
**SCQ**	x							
**Spence PAS**	x	x	x	x	x	x	x	x
**MAP-DB**	x						x	x
**Conners’**	x						x	x
**CBCL-Parent**	x							x
**CBQ**	x							
**BRIEF-P**	x						x	
**BIQ**	x						x	
**ERC**	x						x	x
**Homework**		x	x	x	x	x		
**PARENT (ON SELF)**								
**BAI**	x						x	
**PSWQ**	x						x	
**PHQ-9**	x						x	
**PSI-4**	x						x	
**CCNES**	x						x	
**DERS**	x						x	
**ATQ**	x						x	
**TEACHER (ON CHILD)**								
**C-TRF**	x							x
**CHILD (ON SELF)**								
**ESI**	x						x	
**EEG TASK**								
**Zoo task (ERN, ICPS)**	x						x	
**BEHAVIORAL TASKS**								
**HTKS**	x						x	
**NIH Toolbox-Vocab**	x							
**NIH Toolbox-DCCS**	x						x	
**NIH Toolbox-Flanker**	x						x	
**Dinky Toys**	x						x	
**Stroop**	x						x	
**Tongue**	x						x	
**Potential Threat Task**	x						x	
**Free Play**	x						x	
**Perfect Circle**							x	
**Gift Wrap**	x						x	
**Disappointing Toy**							x	
**Speech Task**							x	
**Bubbles Task**							x	

## Data Availability

The datasets generated and/or analyzed during the current study are not publicly available due to participant confidentiality but are available from the corresponding author on reasonable request.
